# Involving patients as research partners exemplified by the development and evaluation of a communication-skills training programme (KOKOS-Rheuma)

**DOI:** 10.1007/s00393-020-00839-7

**Published:** 2020-07-16

**Authors:** A. C. Schöpf-Lazzarino, P. Böhm, U. Garske, M. Schlöffel, A. Stoye, J. Lamprecht, W. Mau, E. Farin

**Affiliations:** 1 Section of Health Care Research and Rehabilitation Research, Faculty of Medicine and Medical Centre, University of Freiburg, Hugstetter Str. 49, 79106 Freiburg, Germany; 2 Division of General Practice/Family Medicine, Faculty of Medicine and Medical Centre, University of Freiburg, Freiburg, Germany; 3 Deutsche Rheuma-Liga Bundesverband e. V., Bonn, Germany; 4 Institute of Rehabilitation Medicine, Interdisciplinary Centre for Health Sciences, Medical Faculty, Martin-Luther-University Halle-Wittenberg, Halle (Saale), Germany

**Keywords:** Patient participation, Education, Communication, Rheumatic diseases, Musculoskeletal diseases, Patient*innenbeteiligung, Ausbildung, Kommunikation, Rheumatische Erkrankungen, Muskuloskeletale Erkrankungen

## Abstract

Despite widespread recommendations for involving patient research partners (PRPs), there is little information about how patients have been involved in research. Our aim was to describe and assess the contributions of four PRPs in a project on communication-skills training funded by Deutsche Rheuma-Liga Bundesverband e. V. (German League Against Rheumatism [GLR] is a patient organisation for people with rheumatic and musculoskeletal diseases). The PRPs’ participation was beneficial with regards to content and organisation. Thanks to their participation, we could enlarge our sample by over a third, and they contributed their own ideas to the training. Four PRPs added their perspective of various regional organisations. Outside this project, they were also very active within GLR and experienced in managing their rheumatic disease. To achieve more representativeness, future studies might also employ strategies to engage individuals with less experience in dealing with their disease, e.g. newly diagnosed patients. While the collaboration between PRPs and researchers proved very successful, more regular discussions about tasks and responsibilities would be worthwhile.

## Abstract (de)

Obwohl die Einbeziehung von Patient*innen als Forschungspartner*innen (FP) in wissenschaftlichen Forschungsprojekten weithin befürwortet wird, gibt es bisher wenig Erfahrungsberichte über die konkrete Umsetzung partizipativer Forschungsprojekte. Dieser Beitrag beschreibt die Mitarbeit von 4 FP im Rahmen eines von der Deutschen Rheuma-Liga Bundesverband e. V. (DRL) geförderten Projekts zur Entwicklung einer Kommunikationsschulung und bewertet die Mitarbeit systematisch. Die Mitwirkung der FP ging mit inhaltlichen und organisatorischen Vorteilen einher: So konnte die Teilnehmerzahl um über ein Drittel erhöht werden. Außerdem ergänzten die FP die im Projekt entwickelte Schulung um eigene Ideen. Die 4 FP brachten ihre Erfahrungen in Bezug auf verschiedene Landesverbände ein. Sie waren unabhängig vom Projekt sehr engagiert in der DRL und hatten viel Vorerfahrung bezüglich des Umgangs mit ihrer jeweiligen rheumatischen Erkrankung. Zur Erreichung einer besseren Repräsentativität sollten künftige Studien versuchen, auch neu erkrankte und somit in Bezug auf das Krankheitsmanagement unerfahrenere Patient*innen zu beteiligen. Obwohl sich die Zusammenarbeit zwischen den FP und den Forschern als sehr erfolgreich erwies, wird ein regelmäßigerer Austausch über Aufgaben und Verantwortlichkeiten als vorteilhaft angesehen.

While there are indications that participatory research is beneficial, the empirical evidence on its effects is limited. Moreover, there is little descriptive information about how patient research partners have been involved in projects. The aim of this paper is thus to systematically describe our experiences involving patients in a project on communication-skills training for people with rheumatic and musculoskeletal diseases.

Participatory research is not a specific research methodology. One can regard it as the approach of including the stakeholders of research and of appreciating their engagement in the research process instead of viewing them only as research subjects [5]. In healthcare research, stakeholders can be patients, healthcare professionals and health insurance providers and others. Patients and the public are the most frequent groups [7]. Participatory research differs in relation to its approach to engaging patients.^1^ A widely used continuum by Hanley et al. [14] extended by Sweeney and Morgan [23] comprises four approaches: “consultation”, “contribution”, “collaboration” and “control”. *Consultation* means that patients are asked for their opinions, but researchers need not act on them. For instance, in relation to research agenda-setting, patients with diseases such as ulcerative colitis [26], or asthma and COPD [6] were interviewed to identify and prioritise research topics. *Contribution* signifies an active involvement of patients without them having any decision power. These could be patients applying their experience and background knowledge to analyse data without having the power to influence wider decisions [23]. This contrasts with *collaboration*, in which patients and researchers share the decision power. In a study by Belam et al. [3] on the suffering of patients with migraines, researchers and patients made conjoint decisions about the interview process and data analysis. In the CREST.BD studies investigating psychosocial factors in bipolar disorder, patients worked collaboratively with researchers at various tasks such as developing interview schedules, analysing data, developing a quality-of-life scale and disseminating results [17]. When patients are in *control*, they have the power to make decisions. Walsh and Boyle [25], having experience in mental health issues, conducted a focus group study about the coping strategies of psychiatric inpatients and how hospital services can support patients in their recovery. The authors received training and support from researchers and peers over a 12-month period.

Any of these approaches can be taken in a research project, but they can also vary between different phases of the same study. There are different proposals for dividing research phases in participatory research. Cargo and Mercer [5] used three broad phases: “shaping the purpose and scope of the research”, “research implementation and context” and “interpretation and application of the research outcomes”. Shippee et al. [22] suggested three slightly different phases. Their framework is based on a systematic review on patient and service-user engagement and consists of three phases—preparatory phase, execution phase and translational phase—which are differentiated further into eight subphases or stages.

The literature contains recommendations to support participatory research across all phases of a study. Based on a systematic review, a Delphi method and a survey, de Wit et al. [8] derived eight recommendations (R1–R8), i.e. patients’ participation should be considered for all research projects (R1) and in all phases (R2). So far, patient participation has been particularly reported in agenda-setting and study design [9]. Moreover, at least two patients should be involved (R3) to strengthen the impact of the patient perspective and to deal effectively with any temporary or permanent dropouts. During the process of recruiting and selecting patients, the expected contributions should be specified to avoid disappointment (R4), and the skills and motivation of potential candidates should be considered (R5). It is also recommended that the principal investigator creates an atmosphere that supports patient contributions (R6) and ensures that they are equipped for their tasks (R7) (e.g. by providing training). However, this does not mean the aim is that patients become “professional researchers”. Lastly, the patients’ contributions need to be acknowledged (R8), also meaning co-authorship when justified.

The benefits of participatory research might depend on the interplay between study phase and participation approach. Research can profit from the inclusion of patients in particular by highlighting the patient perspective [4, 10]. Cargo and Mercer [5] listed potential benefits of participatory research for researchers and nonresearchers. For example, researchers may benefit as they can achieve deeper understanding of a health issue; recruitment and response rates can be higher; and they might be able to disseminate research findings to more diverse audiences. Participatory research can empower nonresearchers and provide them with new knowledge and skills. These benefits depend on the actual implementation of participatory research. Problems can occur because of patients’ loss of interest in the project [24], time restrictions, power imbalances and other factors [5].

## Project KOKOS-Rheuma

**Fig. 1:**
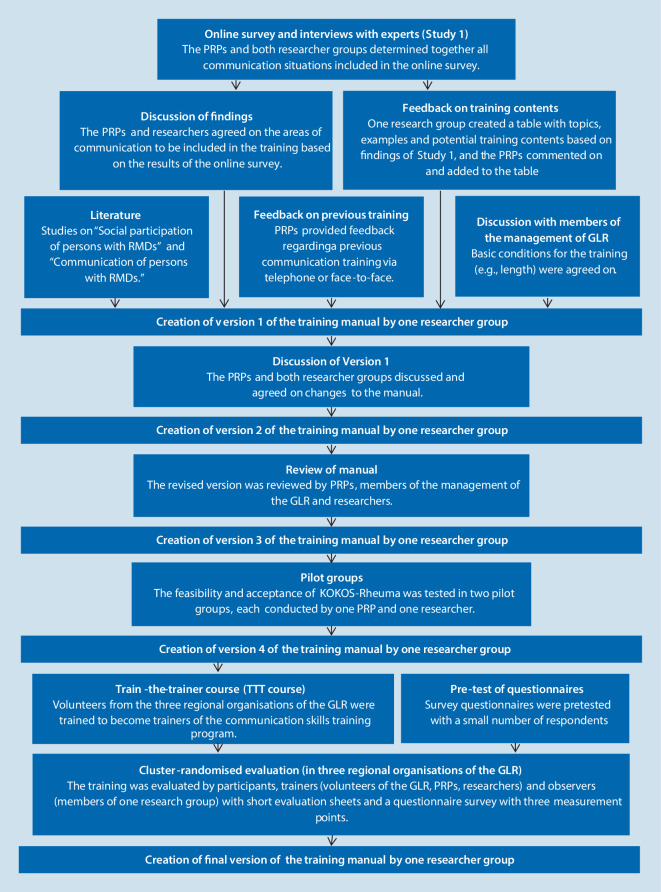
Stages of the development and evaluation of KOKOS-Rheuma. *PRP* patient research partner, *RMDs* rheumatic and musculoskeletal diseases, *GLR* German League against Rheumatism, *TTT course* Train-the-trainer course

Our project consisted of two studies.^2^ In Study 1 we identified factors influencing disease-related communication and the social participation of people with rheumatic and musculoskeletal diseases [16]. For this purpose we conducted an online survey of persons with rheumatic and musculoskeletal diseases and interviews with experts. In Study 2, we developed and evaluated a training programme for individuals with rheumatic and musculoskeletal diseases to improve their communication skills in various everyday situations [20]. We evaluated the training programme by having the participants, trainers and observers fill out evaluation forms (formative evaluation) [20] and performed a questionnaire study with three measurement points (summative evaluation). Fig. 1 shows the main research steps. The ethics committees of Martin-Luther-University Halle-Wittenberg and University of Freiburg approved the study.

This project was funded by Deutsche Rheuma-Liga Bundesverband e. V. (German League Against Rheumatism [GLR]). The GLR is one of the largest patient organisations in Germany and comprises a national organisation and regional ones for the federal states. These are broken down further into local organisations and individual self-help groups.

## Patient research partners

The European League Against Rheumatism (EULAR) as well as the GRL advocate participatory research by incorporating patient research partners. According to EULAR’s task force developing recommendations for including patient research partners (PRPs) in EULAR-funded scientific projects, PRPs are “persons with a relevant disease who operate as active research team members on an equal basis with professional researchers, adding the benefit of their experiential knowledge to any phase of the project” [8]. This definition best reflects the collaboration approach introduced [14, 23].

Four GLR members of three geographically separated GLR regional organisations participated in our project. Two of them attended a preparatory course (lasting 2 days) taught by the GLR. The PRPs were introduced to topics such as the types of scientific studies, how to phrase research questions and hypotheses, searching for scientific publications, different research methods and approaches, participative research, and how to assess research proposals. These PRPs were assigned by the GLR after the funding decision. The researchers had already sought PRPs while drafting the project proposal. The first author asked participants in a previous communication-skills training programme [21] whether they were interested in participating in the present project.

## Methodology

The first step was to systematise the PRPs’ tasks according to the Shippee et al. [22] framework’s phases in order to provide a complete and systematic overview of their participation. An approach to participation as described by Sweeney and Morgan [23] was assigned to each stage according to the PRPs’ tasks.

## Findings

### Preparatory phase

The preparatory phase entails the “agenda setting” and “funding” stages. The former addresses identifying and prioritising research topics, and the second is about the allocation of funds. Choosing the topic of the call for proposals and the decision about funding were not part of the project. The level of participation is therefore unknown. However, the call for proposals was made by one of the largest patient organisations in Germany; they requested that at least two PRPs be involved in the project. The fact that the PRPs’ involvement was obligatory is an important step in strengthening participatory research.

### Execution phase

The execution phase consists of four stages. The “study design and procedures” stage entails the decision about how the research should be done. In our study, two PRPs were involved in this stage. After discussing with them whether the topic of creating a communication-skills training programme specifically for rheumatic patients was relevant and worthwhile, the researchers prepared a project proposal outlining the study design and involvement of the PRPs. As the two PRPs provided feedback, their involvement can be classified as *consultation*.

The research participants are enrolled in the “recruitment and participation” stage. Here, the PRPs revised documents in both studies (information leaflets, informed consent forms, etc.). They actively engaged in recruiting participants by distributing the link to the online questionnaire and information about the training via e‑mail and at events. Moreover, we were only able to complete the cognitive pretests of the newly developed questionnaires (later required to assess the training programme) thanks to a PRP’s input. When the PRPs were informed that too few training participants had been recruited in the originally selected regional organisation, two of them arranged that the training programme also could be carried out in two other regional organisations by recruiting participants and trainers (lay persons). They organised one train-the-trainer course and 5 of 16 training courses in the evaluation phase. As a result, the PRPs increased our sample size by over a third.

In the “data collection” stage, patients can engage in developing measurement instruments and data collection. We also included the intervention’s development and conduct in this stage. At their first meeting, the PRPs and researchers identified which communication situations are relevant for the online survey. The PRPs were involved in developing several items and made suggestions regarding shortening the survey and pretesting the online survey. The PRPs also amended the schedule of interviews with experts. In Study 2, after conjointly deciding on the communication situations to be included in the training module, the researchers prepared a document comprising topics, examples and potential programme content based on responses to the open questions in the online survey and expert interviews. The PRPs commented on the suggestions and included their own ideas. They also informed the researchers in a face-to-face meeting or via telephone about which elements in the previous communication training programme [21] they wanted to keep or delete. The researchers provided an initial draft of the training manual which was discussed later at a meeting. Here, the PRPs also advocated for their own ideas (such as including the topic “Short explanation of one’s own disease” and more examples regarding young rheumatics). A revised second version of the training manual, slides and a newly created participant’s booklet were prepared by the researchers and then reviewed by the PRPs and members of the GLR board and management. The PRPs also carried out both pilot training courses conjointly with a researcher and helped out by conducting four training courses during the evaluation phase with a trained lay person or another PRP. They therefore ensured that those training courses took place.

In the “data analysis” stage, PRPs can assist in analysing and interpreting data. In our study, the PRPs were not involved in any data analyses or the interpretation of interviews with experts and questionnaire survey of the training evaluation. As some research results were delayed, the information flow to the PRPs was temporarily interrupted. However, they were closely involved in interpreting Study 1’s online survey. Once the online survey’s results were presented to the PRPs, they and the researchers discussed and agreed on which main communication situations be included in the communication training module. Relying on Study 2’s participant, trainer and observer evaluation forms, the researchers presented the results and initial suggestions for amendments. All amendments were discussed and decided upon in a conjoint meeting. The final versions of the training programme and training materials were not forwarded to the GRL until after the PRPs had given their consent to the final versions thereof. As in all three “recruitment and participation”, “data collection” and “data analysis” stages, there were shared decisions on matters such as expanding the recruitment area and training content, and in making amendments; the strongest participation level reached here was *collaboration*.

### Translational phase

The translational phase comprises three stages. The “dissemination” stage includes publications in scientific journals, but also in ways more accessible for the target group. The PRPs’ strongest participation was in *control *at this stage. PRPs described their experiences and the project at national and international conferences, journals and on the GLR homepage. These contributions were either initiated by the researchers or by the PRPs themselves, informing the researchers about their intentions. In addition, in Study 2 the researchers explained the process of publishing scientific articles and proposed co-authorship on several scientific publications to the PRPs. Each PRP could decide whether, and if yes, which article he or she would co-author.

The implementation steps and methodology are decided in the “implementation” stage. The PRPs also took control here, as they continued to provide the training programme in two regional organisations.

In the “evaluation stage”, the collaboration between patients and researchers is assessed. The sole evaluation of the participation process took place at the project’s end in form of a final reflection on the participation and collaboration with researchers, as this article describes. While the tasks of the patient research partners and the time schedule were discussed at the beginning of the project, and both sides were open to suggestions about improving the process, there was no continuous evaluation of the work process and cooperation.

## Discussion

Concerning participation, we aimed to achieve patient involvement as described in the aforementioned EULAR task force definition of the ideal patient representative [8]. We thus chose to implement a collaborative approach according to Hanley et al. [14] and Sweeney and Morgan [23]. This we achieved in many but not all project phases and stages. While it is important to discuss whether there are ways to achieve a collaborative approach, one should also keep in mind that increasing participation along the continuum from consultation to control need not necessarily mean that a participatory approach that is more emphatic in this continuum is better than an approach entailing less participation. Some authors argue that participatory research is more about shared learning and partnership than complete control, which falsely suggests that all patients want to have this control. Depending on factors such as interest and requirements of the project stage, many argue that all participatory approaches have their rightful place [19].

### Overall project

Recruiting suitable PRPs is essential for participatory research. Most commonly, convenience sampling takes place, e.g. by recruiting patients in clinics. Also, the patient research partners in this study volunteered by answering a request by the researchers or by applying to the GRL. As a result, our PRPs were highly motivated and experienced in managing their disease, resulting in their profound support of the project. However, one can argue whether they are representative of the majority of our intended target group. For future studies, recruitment strategies attracting other target groups (such as newly diagnosed patients or their relatives) as PRPs should also be considered. By these means, the perspectives and needs of individuals with less experience in managing rheumatic diseases could be taken into account and greater representativeness could be achieved.

Also, an adequate number of PRPs is important to represent the patient perspective and differences in opinion sufficiently [8]. In this project, four patient research partners were involved to represent the needs of people with different rheumatic and musculoskeletal diseases and different regional organisations. This number proved beneficial, as they still revealed different perspectives even if one PRP could not engage in a specific task, and we maintained a good balance between PRPs and researchers. Having enough PRPs can prevent imbalances in power that can hinder PRPs from expressing their own opinion or disagreeing with the researchers [13]. However, involving a higher number of PRPs is inadvisable if team members live as far apart as they do in this study, as regular face-to-face meetings with all team members become difficult and costly.

Another issue is the training of PRPs and researchers to conduct a participatory research project. Two of our PRPs were provided with special training by the GLR. At the beginning of the project, it might have proven beneficial to discuss whether that training had sufficed, or whether additional training for them, the other two patient research partners or researchers would have been desirable. This discussion would have revealed whether the special training enabled the PRPs to become involved in more tasks than they did.

### Preparatory phase

In competitive research funding, it is essential that those who decide about the focus of the call for proposals and funding differ from the persons applying for it. Nonetheless, in this phase, participatory research has been addressed in several studies to identify research priorities and set research agendas (for example, with patients suffering from asthma/COPD or diabetes) [1]. The participation of patients in funding decisions improved, but remains mainly controlled by scientists or administrators.

### Execution phase

Although the “study design and procedure” stage strongly influences a project, participation is particularly difficult due to the nature of most research funding. Gray et al. [13] emphasise the tension between securing research funding and involving patients in writing the proposal. More traditional funding agencies rejected their proposal, which was not specific enough or well elaborated, as many details depended on shared decisions with the participating self-help groups. Moreover, members of the self-help groups on their part were dissatisfied when the researchers included details to help the proposal more closely resemble common practice. While there are studies that achieved at least some participation when developing research proposals, PRP involvement often only started after researchers had applied for a grant [18]. In our study, only two PRPs were involved in writing our proposal. While their feedback indicated that the topic was relevant and helped us adjust the proposal, more time to exchange ideas and the participation of all four patient research partners might have helped (for example, it would have provided us researchers with better understanding about the GLR’s organisation). One potential consequence is that we would have contacted other regional GLR organisations right from the start. One approach to this problem would involve having funding agencies either provide financial support for PRPs for attending meetings during this phase or allow more flexibility in proposals. To prevent PRPs from being excluded a priori from writing a proposal, it should be clarified which additional knowledge and expertise they need to contribute (i.e. by discussing the relevance of the research topic or providing insights about the acceptance of certain research methods in the community).

As other studies have reported [5], our patient research partners played a key role at the recruitment stage by increasing the number of participants, and it was a real advantage that they belonged to different regional organisations. However, this was also a disadvantage, as only a few personal meetings with all project members present could be held. While the patient research partners were involved in all major decisions regarding the training and provided feedback on all training versions, more regular personal meetings and probably more practicable phone/video conferences could have enabled us to involve them in more basic decisions right from the beginning (such as deciding on the manual’s phrasing).

In the data analysis stage, the PRPs in this study were involved in most interpretations of results but not in the analyses themselves. As de Wit et al. [8] state, the aim of participatory research is not to turn PRPs into professional researchers. Therefore, it might make sense to not involve them in all analyses, as some of the latter require very specialised knowledge and expertise. There are examples in which PRPs have been involved in data analysis (such as analysing qualitative data from interviews) [2, 18], but in many cases, PRPs have been excluded from the analysis [13, 24]. In this study, we researchers did not involve the PRPs in some tasks because we thought they were our responsibility and due to time restrictions. Here, it is advisable to discuss forthcoming tasks and time schedules on a regular basis and not hastily presume that some tasks are perforce only the responsibility of either the PRPs or researchers.

During the “translational” phase, PRPs should be invited to become co-authors of publications if they qualify, and not just to avoid the awful publication practice of “ghost writers” [12], but also to acknowledge their work and contributions [8]. Yet involving PRPs is not merely a way to appreciate their contribution—it can also benefit the project. They can contribute to or initiate presentations and publications, and translate them for lay audiences. They can thereby increase the degree of dissemination [10], which also happened in our study.

For the “implementation” stage, we hypothesised that patient participation would positively influence the dissemination to patients and also to changes in the healthcare setting. However, Esmail et al. [10] reported on no qualitative or quantitative assessments of these factors. In our study, we observed a positive impact from involving our PRPs, as they had already initiated and conducted further training courses on their own. A potential task for the future is to conduct future train-the-trainer courses to ensure the continuation of the training programme and extension to other regional organisations.

While the literature claims the many advantages of participatory research, few studies have actually evaluated participation’s impact. Published investigations mostly measured short- but not long-term effects of participation [10]. In our project we did no ongoing systematic evaluation of the relationship, but as this article shows, our project confirmed many of the positive impacts of PRPs other studies reported [10]. However, as stated earlier [7, 11], more time is needed to establish a fruitful working relationship and involve patient research partners more closely in the process. Regular discussions about and evaluations of the quality of collaboration might have helped to determine whether some of our PRPs would have liked to be involved in additional tasks. It might have also been beneficial to include the GLR in discussing the roles of different parties and communication pathways.

## Conclusions for research practice

- Engaging PRPs proved to be beneficial with regard to content and organisation.
- Future studies might benefit from employing strategies to engage individuals with less experience in dealing with their disease to achieve greater representativeness.
- It could prove beneficial to set up more regular discussions about the tasks and responsibilities of all persons involved in a participatory project.

## Footnotes

1 Hayes et al. [15] argued for abandoning the term “level” using “approach” instead, as research projects can use a combination and as there are no clear-cut boundaries.

2 Both studies were registered in the German Clinical Trials Register (Study 1 = DRKS00008323 and Study 2 = DRKS00008054).
